# Circ_0000705 facilitates proline metabolism of esophageal squamous cell carcinoma cells by targeting miR-621/PYCR1 axis

**DOI:** 10.1007/s12672-022-00513-1

**Published:** 2022-06-22

**Authors:** Cui-juan Qian, Yi-yang Tong, Lin-ken Wu, Yi-chao Wang, Xiao-sheng Teng, Jun Yao

**Affiliations:** 1 Early Gastrointestinal Cancer Research Center, Taizhou Central Hospital (Taizhou University Hospital), Taizhou University, Taizhou, 318000 Zhejiang Province China; 2 School of Medicine, Taizhou University, Taizhou, 318000 Zhejiang Province China; 3 Department of Medical Laboratory, Taizhou Central Hospital (Taizhou University Hospital), Taizhou University, Taizhou, 318000 Zhejiang Province China; 4 Department of Gastroenterology, Taizhou Central Hospital (Taizhou University Hospital), Taizhou University, Taizhou, 318000 Zhejiang Province China

**Keywords:** ESCC, Proline metabolism, Circ_0000705, miR-621, PYCR1

## Abstract

CircRNAs have been found to play crucial roles in the metabolism and progression of cancers, but their roles and mechanisms in esophageal squamous cell carcinoma (ESCC) have not been fully elucidated. This work is aimed to explore the role and mechanism of hsa_circ_0000705 (circ_0000705) in ESCC. Circ_0000705 expression was up-regulated in ESCC tissues and cell lines, and high circ_0000705 expression was correlated with poor survival. Circ_0000705 facilitated cell proliferation, invasion, migration and proline metabolism of ESCC cells. The inhibitory effects of circ_0000705 knockdown on cell invasion, migration and proline metabolism were partly rescued by miR-621 inhibition or PYCR1 over-expression. Furthermore, circ_0000705 expression is negatively correlated with miR-621 expression, and positively correlated with PYCR1 in ESCC tissues. Mechanistically, circ_0000705 acted as a ceRNA by sponging miR-621, thereby facilitating PYCR1 expression in ESCC cells. In conclusion, circ_0000705 promoted proline metabolism and malignant progression of ESCC by regulating the miR‑621/PYCR1 axis.

## Introduction

Esophageal cancer (EC) is the eighth most common cancer and sixth most deadly cancer in the world [1, 2]. The main pathological type of EC is esophageal squamous cell carcinoma (ESCC), which accounts for about 90% of all ECs [3, 4]. Despite increasing advances in the diagnosis and treatment of ESCC in recent years, the overall survival rate (OSR) in ESCC patients has not markedly elevated over the last decade [5, 6]. Because of the lack of specific early symptoms and diagnostic markers, most ESCC patients are diagnosed at an advanced stage, which leads to high mortality in ESCC patients [6, 7]. Therefore, to clarify the molecular mechanism of the malignant progression of ESCC, and find new biomarkers and targets, are of great significance for ESCC diagnosis, treatment and prognosis.

Increasing researches have manifested that non-coding RNAs (ncRNAs), including circular RNAs (circRNAs), microRNAs (miRNAs) and long non-coding RNAs (lncRNAs), participate in the regulation of DNA replication, transcription, translation and epigenetic modification of oncogenes, tumor suppressor genes and other genes in a variety of cancers [8]. It’s worth noting that circRNAs can act as competing endogenous RNAs (ceRNAs) to sponge their corresponding miRNAs and thus modulate their downstream target genes’ expressions in cancers [9]. Moreover, increasing studies reported that some circRNAs have been identified as key regulators in ESCC progression, and some dysregulated circRNAs are related to the outcomes of ESCC patients [10–12]. Therefore, it is feasible that some circRNAs can be used as diagnostic and prognostic biomarkers or/and therapeutic targets for ESCC.

Proline metabolism, which synthesizes and catabolizes proline to generate ATP, is a key feature of metabolic reprogramming in some cancers [13–15]. Although proline metabolism is less efficient in ATP production, it facilitates cell proliferation, restrains cell apoptosis, and produces metabolites to promote cancer cell survival under various stressful conditions [16, 17]. However, the role and mechanism of proline metabolism in ESCC progression remain to be explored. As reported, some circRNAs are involved in the metabolisms of glutamine, serine and other amino acids, and function as crucial regulators in the occurrence and development of some cancers [18–20]. However, whether circRNAs are involved in the regulation of proline metabolism, and the exact relationship between proline metabolism and the malignant phenotype of ESCC need to be elucidated.

Circ_0000705 (hsa_circ_0000705; https://circinteractome.nia.nih.gov/), located on human chr16:58,593,707–58,594,266, is a poorly conserved ncRNA with a spliced seq length of 559 bp. However, so far, there is no research report on circ_0000705 in cancers. In the present study, we found that circ_0000705 was up-regulated and exerted a tumor-promoting effect in ESCC. Mechanistically, circ_0000705 could act as ceRNAs for miR-621 to modulate proline metabolism and malignant phenotype of ESCC cells. Thus, exploring its role and mechanism of circ_0000705 in ESCC can provide novel biomarkers and/or targets for ESCC diagnosis, treatment and prognosis.

## Materials and methods

### Clinical samples

Our clinical study was approved by the Ethics Committee of Taizhou University Hospital. Forty ESCC patients at Taizhou University Hospital were recruited and signed informed consent voluntarily. All patients did not receive radiotherapy or chemotherapy prior to surgical resection. ESCC tissues and their adjacent non-tumor tissues were immediately frozen in liquid nitrogen.

### Cell culture and cell transfection

Human esophageal squamous epithelial cell line (Het-1A) and human ESCC cell lines (KYSE150, KYSE450, KYSE510 and KYSE30) were obtained from the American Type Culture Collection (ATCC, Manassas, VA, USA). All cells were incubated in RPMI 1640 (Gibco, NY, USA) mixed with 10% fetal bovine serum (FBS; Gibco, USA) in an atmosphere at 37 ℃ with 5% CO_2_.

Circ_0000705 or PYCR1 over-expression vectors and circ_0000705 shRNAs were constructed by Geneseed (Guangzhou, China). miR-621 mimic and miR-621 inhibitor were provided by GenePharma (Shanghai, China). KYSE150 and KYSE30 cells were cultured to 70–80% confuence before cell transfections. All the cell transfections were performed using Lipofectamine 2000 (Thermo Fisher Scientific, Waltham, MA, USA) according to the manufacturer’s instructions.

### Quantitative real-time polymerase chain reaction (qRT-PCR)

Total RNA was extracted from ESCC cells or tissues using Trizol reagent (Invitrogen, Carlsbad, CA, USA). Following the determination of RNA concentration and purity, the total RNA was converted to cDNA using the PrimeScript 1st Strand cDNA Synthesis Kit (Takara, Dalian, China) or the Mir-X™ miRNA First-Strand Synthesis Kit (TaKaRa) according to the manufacturer’s instructions. The obtained cDNA was then analyzed by qRT-PCR analysis using SYBR premix Ex Taq™ II kit (Takara) or Mir-X™ miRNA qRT-PCR TB Green Kit (Takara) according to the manufacturer’s requirements. Human GAPDH and U6 genes were used as internal controls. All primer sequences designed using Primer-Blast online tool (https://www.ncbi.nlm.nih.gov/tools/primer-blast/) or as described previously [22, 23] are listed in Table 1. Finally, the relative RNA expression levels were evaluated using the 2^−ΔΔCt^ method.

**Table 1:** qRT-PCR primer sequences

Name	Primer sequence
circ_0000705	Forward: 5′-AACAGTTTTGCCTCAGCCCT-3′ Reverse: 5′-TGGTAACGCCCAGTGCCATA-3′
miR-621	Forward: 5′-GGCTAGCAACAGCGCTTACCT-3′ Reverse: the mRQ 3’ Primer supplied with the kit
PYCR1	Forward: 5′-AAGATGCTGCTGCACTCAGA-3′ Reverse: 5′-CACCTTGTCCAGGATGGTCT-3′
GAPDH	Forward: 5′-GCACCGTCAAGGCTGAGAAC-3′ Reverse: 5′-GCCTTCTCCATGGTGGTGAA-3′
U6	Forward: 5′-GCTTCGGCAGCACATATACTAAAAT-3′ Reverse: 5′-CGCTTCACGAATTTGCGTGTCAT-3′

### Cell counting kit-8 (CCK-8) assay

The CCK-8 kit (Abcam, Cambridge, MA, USA) was used following the manufacturer’s introductions. In detail, ESCC cells (3 × 10^3^/200 μl/well) were seeded into the 96-well plates, and six replicates for each sample were set at the same time. Different intervention factors and time were applied to the cells, and then 10 μl of CCK-8 solution which was evenly mixed in 100 μl of fresh medium were added into each well. Plates were incubated for 2 h at 37 ℃, and the absorbance (A value) at the wavelength of 450 nm was recorded with a microplate reader (Bio-Rad Laboratories, Inc., Hercules, CA, USA).

### Cell migration and invasion assay

For transwell migration test, ESCC cells in logarithmic growth phase were collected in RPMI 1640 containing 1% FBS, and 250 μl cell suspension containing 5 × 10^4^ cells was added into the transwell chamber, and 500 μl RPMI 1640 containing 15% FBS was added into the lower part of the chamber. After incubation for 24 h, the cells that did not migrate in the upper cavity were removed with cotton swabs. The cells at the bottom surface of the transwell chamber were fixed with methanol and stained with DAPI (2.5 µM; Abcam, Cambridge, MA, USA) for 30 min. Then, the stained cells were observed and photographed with fluorescence microscope (200× , Eclipse 80i; Nikon Corporation, Tokyo, Japan). The cells were counted in five random fields, and finally the average was taken.

In the transwell invasion test, the Matrigel was placed in the upper insertion chamber and hydrated for 30 min at room temperature. 1 × 10^5^ cells in RPMI 1640 containing 1% FBS were added into the transwell chamber. After 24 h of incubation, the invaded cells were fixed with 70% ethanol and stained with 0.1% crystal violet for 30 min. Then the membrane with 8 μm pores containing stained invaded cells at the bottom surface was completely cut and dissolved with 200 μl of lysis reagent (33% acetic acid). Finally, 100 μl of lysate was transferred to a 96-well plate, and the absorbance value was measured at 560 nm with a quantitative microplate reader (550; Bio-Rad, USA).

### Reactive oxygen species (ROS), proline and ATP assays

The proline metabolism in ESCC cells was analyzed via ROS, ATP and proline assays as detailed previously [24, 25]. After transfection, the ESCC cells was collected, and then the amounts of ROS were detected via the Reactive Oxygen Species Assay Kit (Beyotime Institute of Biotechnology, Shanghai, China) following the instructions, the amounts of proline were detected with the proline assay kit (Tongwei Biotechnology, Shanghai, China) following the instructions, and the amounts of ATP were detected with the ATP Assay Kit (Beyotime). The relative levels of ROS, proline and ATP were normalized to the control group.

### Pull-down assay with biotinylated miR-621

48 h after transfection with biotinylated wild-type (WT) miR-621 (Bio-miR-621-WT), mutant (MT) miR-621 (Bio-miR-621-MT) or antagonistic miR-621 probe (GenePharma, Shanghai, China), the ESCC cells were collected and lysed in specific lysis buffer (Ambion, Austin, TX, USA) for 10 min, and then mixed with M-280 streptavidin magnetic beads (Sigma-Aldrich, St. Louis, MO, USA) at 4 ℃ for 3 h. TRIzol reagent (Invitrogen, CA, USA) was used to elute and purify the interacting RNA complexes, and qRT-PCR was used to detect the expression levels of circ_0000705.

### Luciferase reporter assay

In order to generate wild-type circ_0000705 reporter gene (circ_0000705-WT) or PYCR1 reporter gene (PYCR1-WT), the partial sequence of circ_0000705 or PYCR1 3′-untranslated region (UTR), which contained the putative miR-621 binding site, was amplified by PCR and then constructed into the pmirGLO luciferase vector (Promega, Madison, WI, USA). Mutant circ_0000705 (miR-621 target site-mutation circ_0000705, circ_0000705-MUT) reporter gene and mutant PYCR1 (miR-621 target site-mutation PYCR1 3′-UTR, PYCR1-MUT) reporter gene were produced by GeneArt™ Site-Directed Mutagenesis System (Thermo Fisher Scientific, Waltham, MA, USA). All constructs were verified by DNA sequencing (Sango Biotech, Shanghai, China)). Subsequently, the luciferase reporter genes and miR-621 mimic or a control mimic were co-transfected into the ESCC cells. The luciferase activity was evaluated at 48 h post-transfection by the dual luciferase reporter system (Promega, Madison, WI, USA) according to the manufacturer’s instructions.

### RNA immunoprecipitation (RIP) assay

The interactions between circ_0000705 and miR-621, miR-621 and PYCR1 mRNA in ESCC cells were detected with the Magna RIP RNA-Binding Protein Immunoprecipitation Kit (Millipore, MA, USA) according to the manufacturer's instructions. In brief, the ESCC cells were lysed in RIP lysis buffer. Then a human anti-AGO2 antibody (dilution, 1:5000; catalog no.ab57113, Abcam, Cambridge, MA, USA) and an IgG control (dilution, 1:5000; catalog no.03–110; Millipore) were conjugated to magnetic beads, which were incubated with the ESCC cell extract at 4 ℃ overnight, respectively. The immunoprecipitated RNA was extracted and subjected to qRT-PCR for the expression levels of circ_0000705 or PYCR1 mRNA, respectively.

### Western blotting

Total cellular protein was extracted from the ESCC cells with RIPA buffer (Beyotime, Shanghai, China), and the protein concentration was determined using BCA Protein Assay kit (Pierce Rockford, IL, USA) following the manufacturer’s introductions. The protein samples were subjected to sodium dodecylsulphate-polyacrylamide gel electrophoresis (SDS-PAGE), and then transferred from the SDS-PAGE gel to the PVDF membrane (Millipore, MA, USA), which was then incubated overnight at 4 ℃ with primary antibody. The primary antibodies against PYCR1 (1:1000, catalog no.ab206693; Abcam, Cambridge, MA, USA) and GAPDH (1:1000, catalog no. ab181602; Abcam) were used. The next day, the PVDF membrane was washed and then incubated with a specific secondary antibody conjugated to horseradish peroxidase for 2 h at room temperature. Finally, immunoreactivity was detected by an enhanced chemiluminescence system kit (Pierce, Waltham, MA, USA) and photographed by an LAS-4000 imaging system (Fujifilm Holdings Corporation, Tokyo, Japan).

### Tissue array

Evaluation of PYCR1 protein expression by immunohistochemistry (IHC) was done on a tissue array (Shanghai Outdo Biotech, Shanghai, China) using an automated immunostainer (Benchmark XT; Roche, Basel, Switzerland), containing 78 paired ESCC tissues and their adjacent normal tissues. The DAKO EnVision system (DAKO, Carpinteria, CA, USA) was used to detect the protein expression of PYCR1. All images (× 200) were photographed and analyzed with an Aperio scanner (Aperio Technologies, Vista, CA, USA). The protein expression of PYCR1 in the tissue array was scored as detailed previously [26].

### Statistical analysis

Statistical analysis was performed using SPSS 25.0 software (SPSS Inc., IL, USA). The results are presented as mean ± standard deviation (SD). Comparisons of two groups were analyzed by Student’s *t*-test, and comparisons of three or more groups were analyzed by one-way analysis of variance (ANOVA) followed by Tukey’s post-hoc test. Survival analysis was performed using the Kaplan–Meier method and analyzed by the log-rank test. *P* values < 0.05 were considered to be statistically significant.

## Results

### Circ_0000705 is highly expressed in ESCC tissues and cell lines

**Fig. 1:**
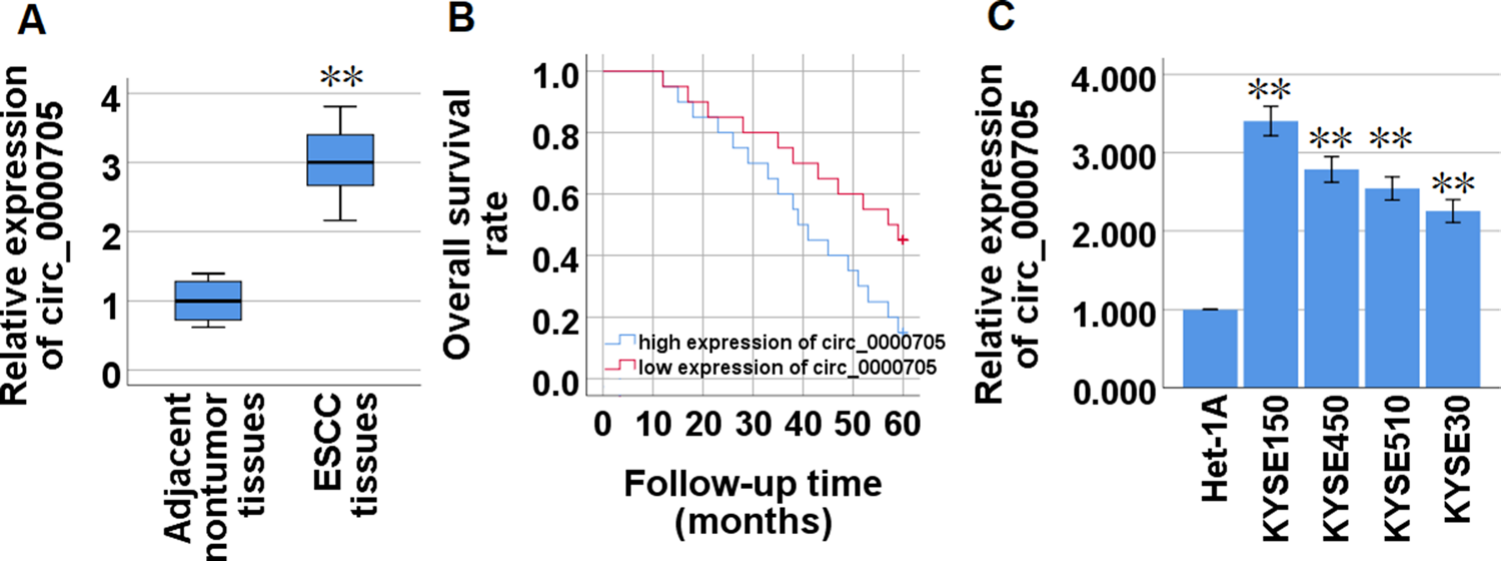
Circ_0000705 expression in ESCC tissue specimens and cell lines. **A** The expression patterns of circ_0000705 in ESCC tissues compared with adjacent non-tumor tissues (n = 40). **B** Kaplan–Meier’s analysis of the correlation between circ_0000705 expression and the overall survival rate (OSR) of ESCC patients. **C** The expression patterns of circ_0000705 in Het-1A and ESCC cell lines (KYSE150, KYSE450, KYSE510 and KYSE30) were detected by qRT-PCR. *P < 0.05, and **P < 0.01 vs control

First, we detected the expression levels of circ_0000705 in 40 paired ESCC and adjacent non-tumor tissues using qRT-PCR, and found that circ_0000705 expression was significantly increased in the ESCC tissues compared to their adjacent non-tumor tissues (Fig. 1A). High expression of circ_0000705 was significantly associated with the lymph node metastasis and TNM stage of ESCC tissues (Table 2). Furthermore, high circ_0000705 expression predicted an unfavourable prognosis (Fig. 1B). Next, we analyzed the circ_0000705 expression level among four human ESCC cell lines (KYSE150, KYSE450, KYSE510 and KYSE30) and one esophageal squamous epithelial cell line Het-1A via qRT-PCR. The present results revealed that circ_0000705 expression was significantly up-regulated in the four ESCC cell lines compared with Het-1A cells (Fig. 1C). In the four ESCC cell lines, KYSE150 cells manifested the highest level of circ_0000705 expression, and meanwhile KYSE30 cells showed the lowest level of circ_0000705 expression (Fig. 1C). Therefore, the two cell lines were used for the next loss- and gain-of-function experiments.

**Table 2:** Relationship between circ_0000705 expression and clinical parameters in 40 clinical ESCC tissue specimens

Clinical parameters	Number of cases	High expression of circ_0000705	Low expression of circ_0000705	*χ*^2^ value	*P* value
Gender					
Male	29	16	13	1.128527	0.288090
Female	11	4	7		
Age					
≥ 60 years old	31	17	14	1.290323	0.255989
< 60 years old	9	3	6		
Differentiation					
Medium and high differentiation	32	15	17	0.625000	0.429195
Poor differentiation	8	5	3		
Lymph node metastasis					
Positive	25	16	9	5.226667	0.022243*
Negative	15	4	11		
TNM stage					
I stage	7	1	6	4.329004	0.037468*
II and III stage	33	19	14		

*P < 0.05

### Circ_0000705 promotes cell proliferation, invasion and migration of ESCC cells

**Fig. 2:**
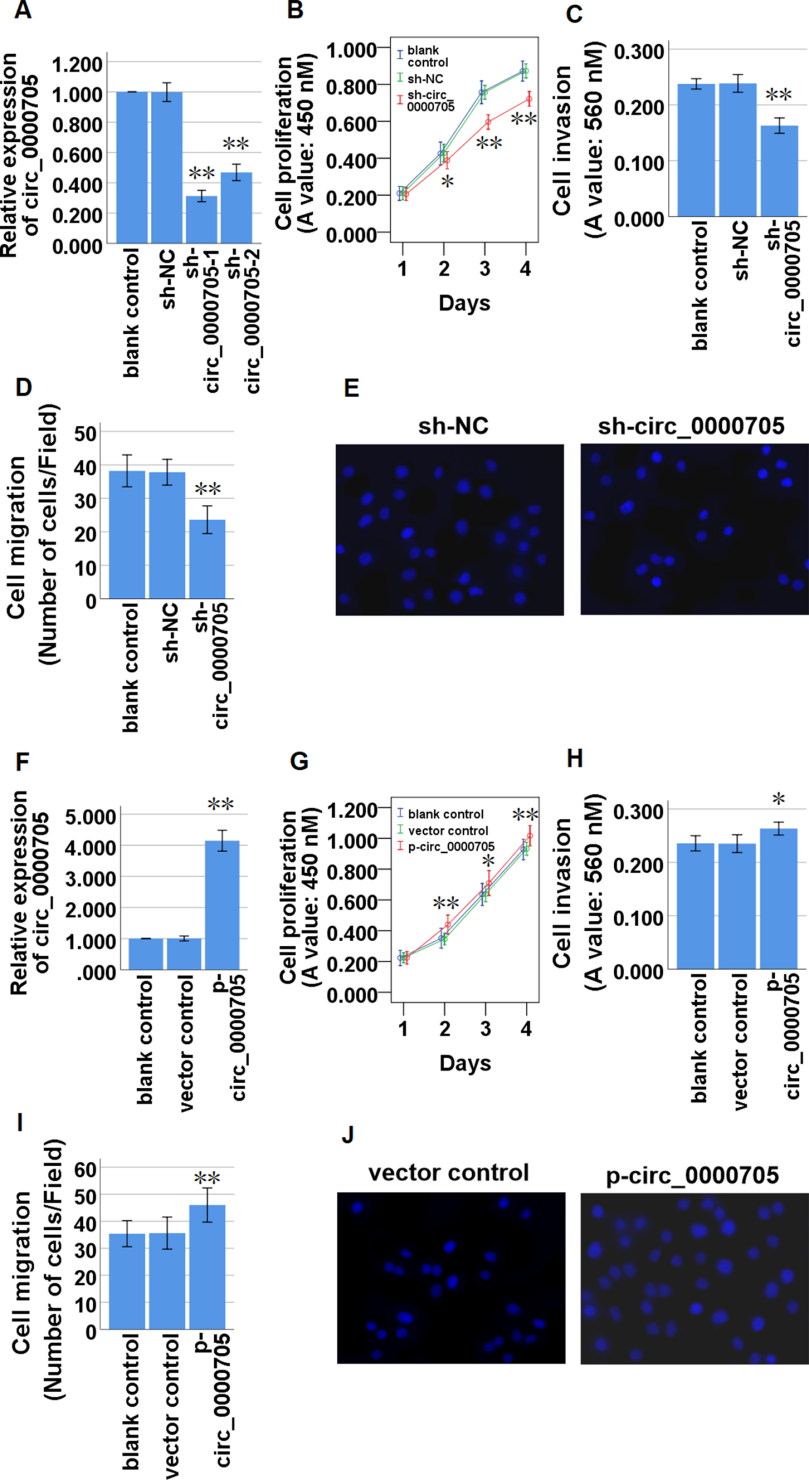
Effects of circ_0000705 on the cell proliferation, invasion and migration of ESCC cells. **A** The expression of circ_0000705 was down-regulated in KYSE150 cells transfected with sh-circ_0000705. **B** Effect of circ_0000705 knockdown on the cell proliferation of KYSE150 cells detected by the CCK-8 assay. **C** Transwell assay with Matrigel was performed to detect the cell invasion in KYSE150 cells with circ_0000705 knockdown. **D** Transwell assay without Matrigel was performed to detect the cell migration in KYSE150 cells with circ_0000705 knockdown. **E** Representative photos of the cell migration in KYSE150 cells were manifested (original magnification, × 200). **F** The expression of circ_0000705 was up-modulated in KYSE30 cells transfected with p-circ_0000705. **G** Effect of circ_0000705 over-expression on the cell proliferation of KYSE30 cells detected by the CCK-8 assay. **H** Transwell assay with Matrigel was used to detect the cell invasion in KYSE30 cells with circ_0000705 over-expression. **I** Transwell assay without Matrigel was used to detect the cell migration in KYSE30 cells with circ_0000705 over-expression. **J** Representative photos of the cell migration in KYSE30 cells were manifested (original magnification, × 200). *P < 0.05, and **P < 0.01 vs control

Two shRNAs against circ_0000705 (sh-circ_0000705-1, sh-circ_0000705-2) were constructed and transfected into KYSE150 cells, the knockdown of circ_0000705 efficiency in KYSE150 cells was evaluated by qRT-PCR, and the shRNA (sh-circ_0000705-1) with the more obvious knockdown effect was chose for the subsequent experiments in KYSE150 cells (Fig. 2A). CCK-8 assay showed that knockdown of circ_0000705 significantly restrained the cell proliferation of KYSE150 cells (Fig. 2B). We further evaluated the effects of circ_0000705 knockdown on cell invasion and migration of ESCC cells. Compared with the control cells, knockdown of circ_0000705 significantly inhibited the cell invasion (Fig. 2C) and migration (Fig. 2D, E) of KYSE150 cells,

The circ_0000705 over-expressing plasmid (p-circ_0000705) was constructed and transfected into KYSE30 cells, and then the over-expression of circ_0000705 efficiency in KYSE30 cells was evaluated by qRT-PCR (Fig. 2F). The CCK-8 assay manifested that over-expression of circ_0000705 significantly facilitated the cell proliferation of KYSE30 cells (Fig. 2G). We further evaluated the effects of circ_0000705 over-expression on cell invasion and migration of ESCC cells. Compared with the control cells, over-expression of circ_0000705 significantly promoted the cell invasion (Fig. 2H) and migration (Fig. 2I, J) of KYSE30 cells.

### Circ_0000705 promotes proline metabolism in ESCC cells

**Fig. 3:**
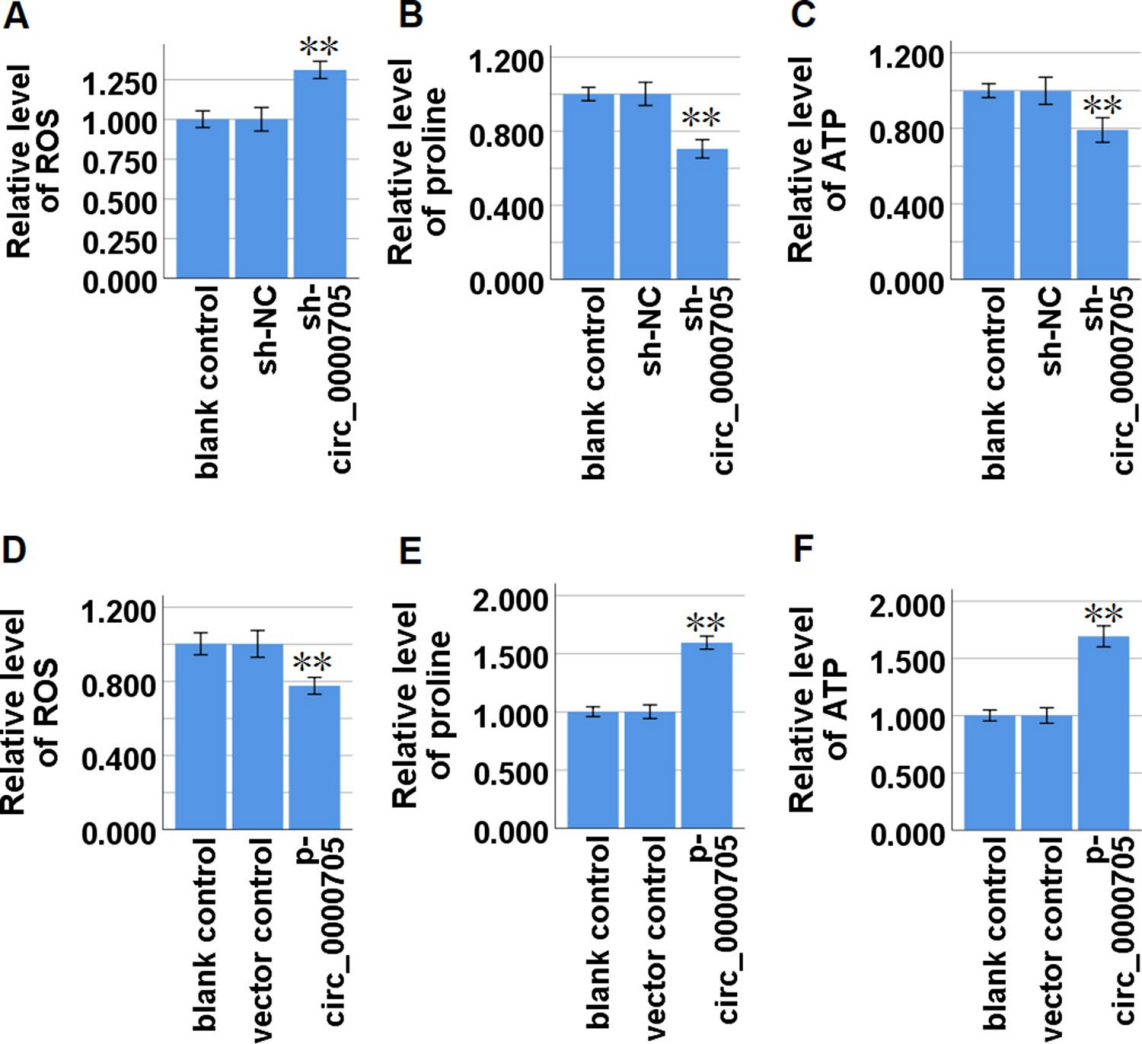
Effect of circ_0000705 on proline metabolism in ESCC cells. **A** ROS level in KYSE150 cells with circ_0000705 knockdown was detected using a corresponding commercial kit. **B** Proline level in KYSE150 cells with circ_0000705 knockdown was detected using a corresponding commercial kit. **C** ATP level in KYSE150 cells with circ_0000705 knockdown was detected using a corresponding commercial kit. **D** ROS level in KYSE30 cells with circ_0000705 over-expression was detected using a corresponding commercial kit. **E** Proline level in KYSE30 cells with circ_0000705 over-expression was detected using a corresponding commercial kit. **F** ATP level in KYSE30 cells with circ_0000705 over-expression was detected using a corresponding commercial kit. *P < 0.05, and **P < 0.01 vs control

To assess whether circ_0000705 contributes to the promotion of proline metabolism in ESCC cells, we used matched assay kits to detect reactive oxygen species (ROS), proline and ATP levels in circ_0000705-knockdown KYSE150 cells and circ_0000705-over-expression KYSE30 cells. Here, we found that circ_0000705 knockdown significantly increased the ROS level (Fig. 3A), and significantly decreased proline (Fig. 3B) and ATP (Fig. 3C) levels in KYSE150 cells. Meanwhile, circ_0000705 over-expression significantly decreased the ROS level (Fig. 3D), and significantly increased proline (Fig. 3E) and ATP (Fig. 3F) levels in KYSE30 cells. Overall, the above findings indicated that circ_0000705 facilitated the proline metabolism in ESCC cells.

### Circ_0000705 acts as a ceRNA by sponging miR-621 in ESCC cells

**Fig. 4:**
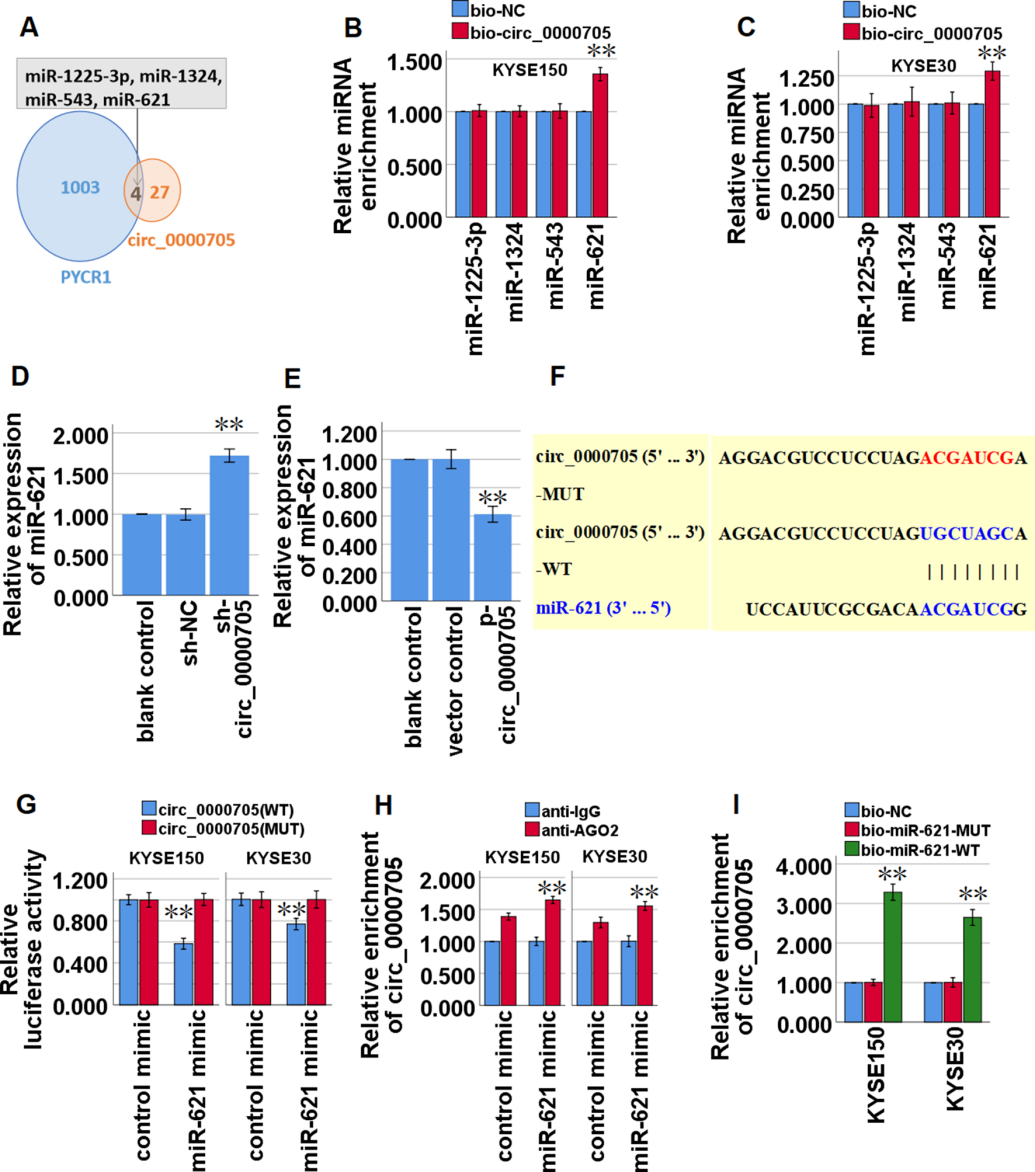
Circ_0000705 binds to miR-621 and inhibits miR-621 expression in ESCC cells. **A** Venn diagram was used to screen the mutual miRNAs for circ_0000705 and PYCR1 mRNA. **B**, **C** The most probable miRNA target was analyzed by RNA pull-down assay with bio-circ_0000705 in KYSE150 and KYSE30 cells. **D** The expression of miR-621 was detected by qRT-PCR in KYSE150 transfected with sh-circ_0000705 or sh-NC. **E** The expression of miR-621 was detected by qRT-PCR in KYSE30 transfected with p-circ_0000705 or the vector control. **F** Bioinformatics analysis suggested that miR-621 might share the binding sites with circ_0000705. The wild-type and the mutated sequences of circ_0000705 (mutation site: red). **G** The luciferase activity of KYSE150 and KYSE30 cells was detected in luciferase reporter plasmid containing wild-type circ_0000705 (circ_0000705-WT) and mutant circ_0000705 (circ_0000705-MUT) co-transfected with miR-621 mimics or negative control (control mimics). **H** AGO2-RIP followed by qPCR to detect circ_0000705 level in KYSE150 and KYSE30 cells after miR-621 over-expression via miR-621 mimic. **I** The cell lysate was incubated with biotin-labeled miR-621, and circ_0000705 expression was measured by qPCR in the products of pull-down by biotin-labeled WT miR-621 or MUT miR-621 in KYSE150 and KYSE30 cells. **P < 0.05, and **P < 0.01 vs control

Through the online predictive analysis of Circular RNA Interactome (https://circinteractome.nia.nih.gov/) and TargetScanHuman (http://www.targetscan.org/vert_72/), it is found that circ_0000705 and PYCR1 mRNA 3′-UTR have the same potential binding sites in some miRNAs. Venn diagram analysis shows that there are four predicted miRNAs (miR-1225-3p, miR-1324, miR-543 and miR-621) that have potential mutual binding sites in both circ_0000705 and PYCR1 mRNA 3′-UTR (Fig. 4A and Tables 3, 4), and RNA pull-down assay demonstrated that, among the four miRNAs in KYSE150 and KYSE30 cells, only miR-621 was captured by circ_0000705 (Fig. 4B, C). To further investigate whether circ_0000705 could regulate miR-621 expression in ESCC cells, we measured the miR-621 expression in circ_0000705-over-expression KYSE30 cells and circ_0000705-knockdown KYSE150 cells, and found that knockdown of circ_0000705 significantly increased the expression level of miR-621 in KYSE150 cells (Fig. 4D), and over-expression of circ_0000705 significantly decreased the expression level of miR-621 in KYSE30 cells (Fig. 4E).

**Table 3:**
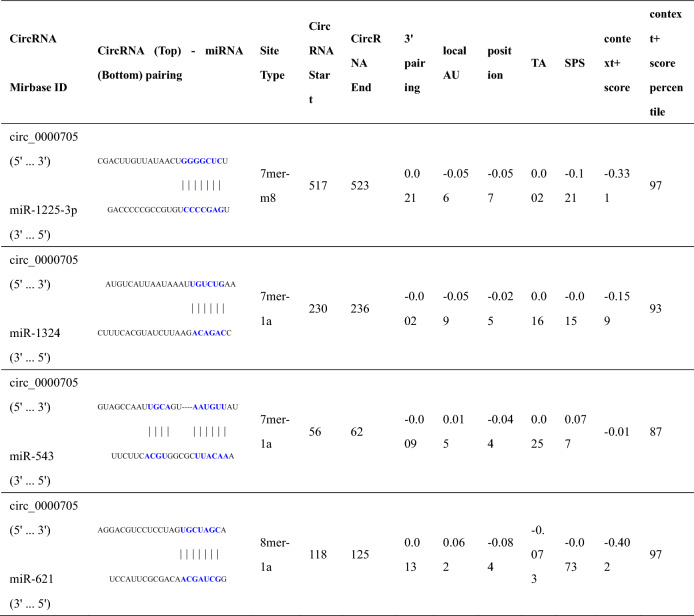
Bioinformatics analysis of the binding between circ_0000705 and the predicted miRNAs

**Table 4:**
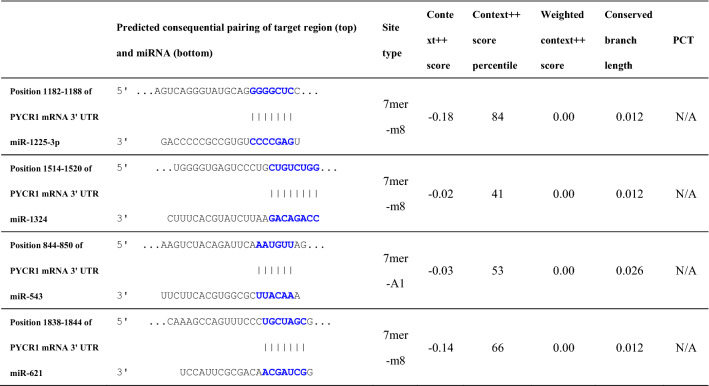
Bioinformatics analysis of the binding between PYCR1 mRNA 3′- UTR and the predicted miRNAs

The binding site between miR-621 and circ_0000705 was exhibited by Circular RNA Interactome (Fig. 4F). Subsequently, a luciferase reporter construct containing circ_0000705 (WT or MUT miR-621 binding site) was generated and co-transfected into KYSE150 and KYSE30 cells with miR-621 mimic. The results of luciferase assay demonstrated that up-regulation of miR-621 expression by transfecting miR-621 mimic significantly inhibited the luciferase activity of WT circ_0000705 but not MUT circ_0000705 (Fig. 4G). To further determine whether circ_0000705 binds miR-621 in an Argonaute 2 (Ago2)-dependent manner, we performed anti-Ago2 RNA immunoprecipitation (RIP) in KYSE150 and KYSE30 cells after transfection with miR-621 mimic, and found that endogenous circ_0000705 enrichment was significantly elevated after up-regulation of miR-621 expression in KYSE150 and KYSE30 cells (Fig. 4H). Furthermore, RNA pull-down assay demonstrated that circ_0000705 bound biotin-labeled the wild type of miR-621 but not mutant miR-621 in KYSE150 and KYSE30 cells (Fig. 4I). Taken together, these above results indicated that circ_0000705 may act as a ceRNA by sponging miR-621 in ESCC.

### miR-621 directly targets PYCR1 mRNA in ESCC cells

**Fig. 5:**
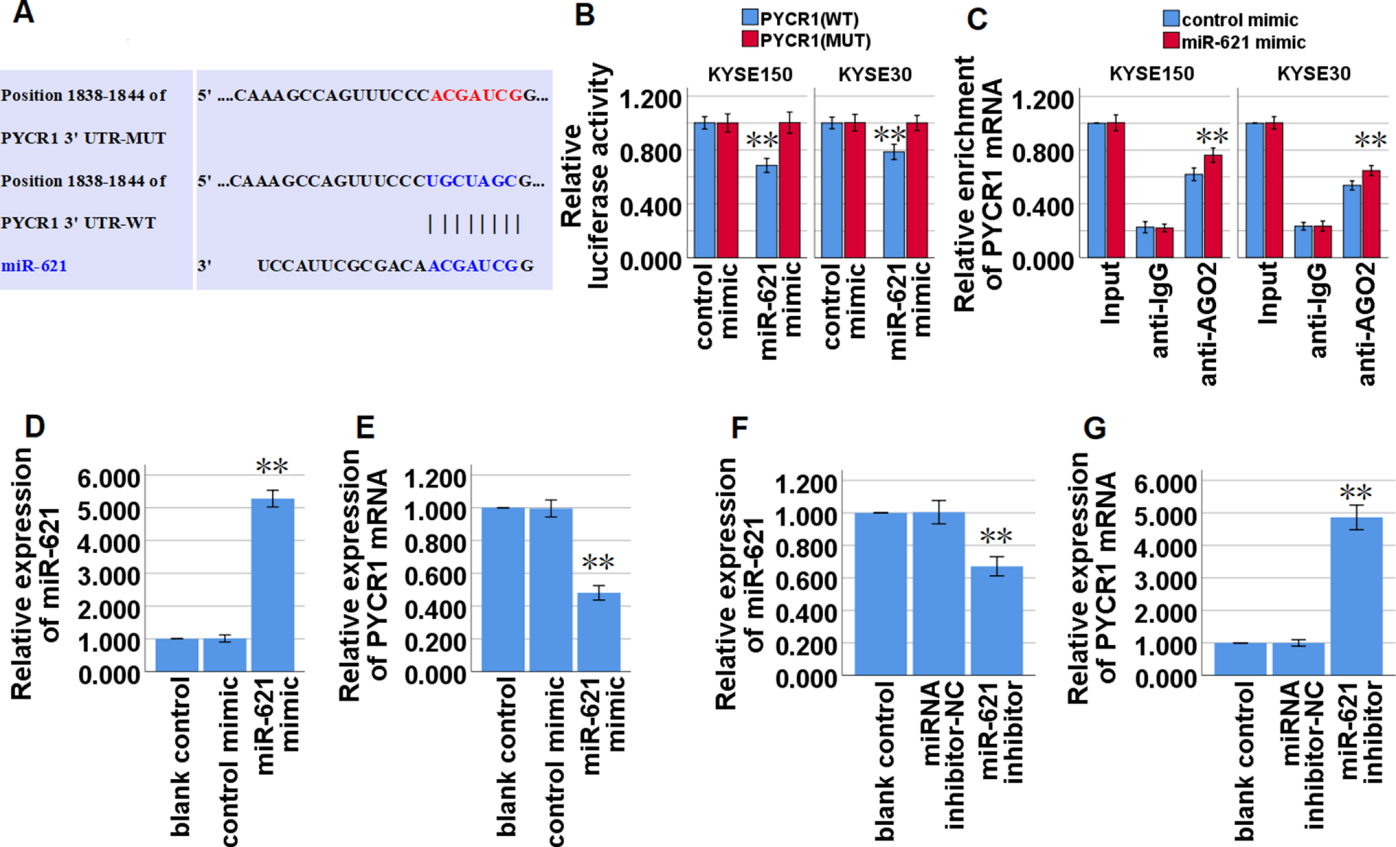
miR-621 directly binds to PYCR1 mRNA 3′ UTR and inhibits PYCR1 expression in ESCC cells. PYCR1 mRNA in ESCC cells. **A** Bioinformatics analysis suggested that PYCR1 mRNA 3′-UTR might share the binding sites with miR-621. The wild-type and the mutated sequences of PYCR1 mRNA 3′-UTR (mutation site: red). **B** The luciferase activity of KYSE150 and KYSE30 cells in luciferase reporter plasmid containing wild-type PYCR1 mRNA 3′-UTR (PYCR1-WT) and mutant PYCR1 mRNA 3′-UTR (PYCR1-MUT) co-transfected with miR-621 mimic or negative control was assessed. **C** RIP assays using antibodies against AGO2 or IgG control were performed in cellular lysates from KYSE150 and KYSE30 cells. qRT-PCR demonstrated the relative enrichment of PYCR1 mRNA in the cells transfected with miR-621 micmic or control mimics. **D** qRT-PCR analysis of miR-621 expression in KYSE150 cells transfected with miR-621 mimic or control mimics. **E** qRT-PCR analysis of PYCR1 mRNA expression in KYSE150 cells transfected with miR-621 mimic or control mimics. **F** qRT-PCR analysis of miR-621 expression in KYSE30 cells transfected with miR-621 inhibitor or miRNA inhibitor-NC. **G** qRT-PCR analysis of PYCR1 mRNA expression in KYSE30 cells transfected with miR-621 inhibitor or miRNA inhibitor-NC. *P < 0.05, and **P < 0.01 vs control

Our present bioinformatics analysis by the TargetScan database (http://www.targetscan.org/vert_72/) showed that miR-621 might share the binding sites with PYCR1 mRNA 3'-UTR (Table 4, Fig. 5A). Dual luciferase reporter assays were used to confirm that miR-621 directly bound to the 3′-UTR of PYCR1 mRNA in KYSE150 and KYSE30 cells, and demonstrated that the miR-621 mimic led to a significant decrease in the luciferase activity of the wild type of PYCR1 mRNA 3′-UTR (PYCR1-WT) reporter, but not in the mutant 3′-UTR of PYCR1 reporter (Fig. 5B). RIP assay demonstrated a higher enrichment level of PYCR1 mRNA in the Ago2 group after up-regulation of miR-621 expression via miR-621 mimic transfection in KYSE150 and KYSE30 cells (Fig. 5C). Furthermore, qRT-PCR analysis demonstrated that up-regulation of miR-621 (Fig. 5D) significantly restrained the expression of PYCR1 mRNA in KYSE150 cells (Fig. 5E), and meanwhile down-regulation of miR-621 expression via transfection with miR-621 inhibitor (Fig. 5F) significantly facilitated the expression of PYCR1 mRNA in KYSE30 cells (Fig. 5G). Taken together, the above data revealed that miR-621 restrained PYCR1 mRNA expression by directly targeting PYCR1 mRNA 3ʹ-UTR.

### miR-621 inhibition or PYCR1 over-expression partly reverses the functions of circ_0000705 knockdown in ESCC cells

**Fig. 6:**
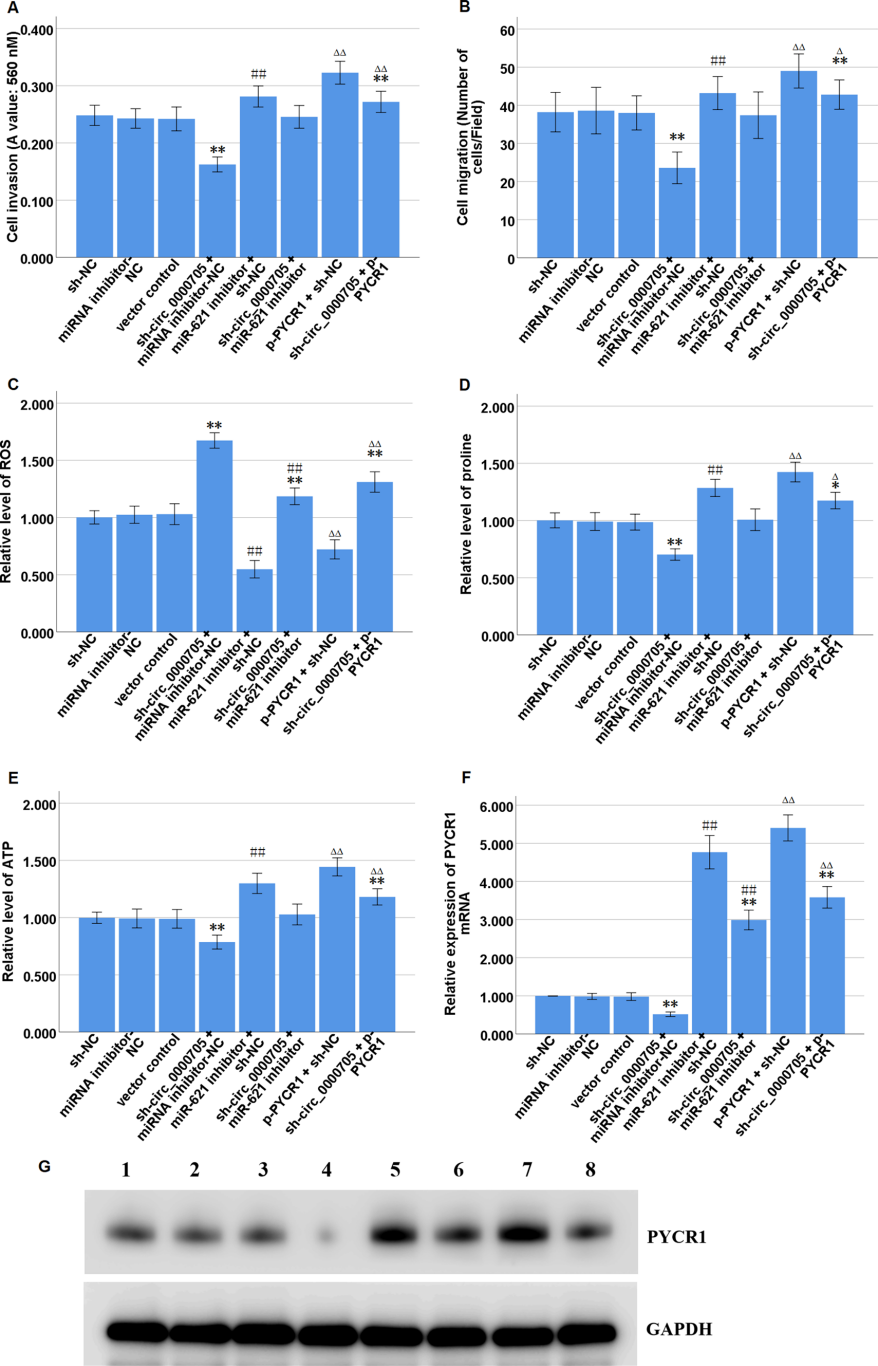
Effects of miR-621 inhibition or PYCR1 over-expression on cell invasion, migration and proline metabolism induced by circ_0000705 knockdown in KYSE150 cells. **A** The transwell assay was performed to detect the cell invasion ability following the transfections in KYSE150 cells. **B** The transwell assay was performed to detect the cell migration ability following the transfections in KYSE150 cells. **C** The commercial kit was used to detect ROS level following the transfections in KYSE150 cells. **D**, **E** The commercial kits were used to detect proline and ATP levels following the transfections in KYSE150 cells. **F** qRT-PCR analysis of PYCR1 mRNA expression following the transfections in KYSE150 cells. **G** Western blot analysis of PYCR1 protein expression following the transfections in KYSE150 cells. Lane 1, sh-NC; Lane 2, miRNA inhibitor-NC; Lane 3, vector control; Lane 4, sh-circ_0000705 + miRNA inhibitor-NC; Lane 5, miR-621 inhibitor + sh-NC; Lane 6, sh-circ_0000705 + miR-621 inhibitor; Lane 7, p-PYCR1 + sh-NC; Lane 8, sh-circ_0000705 + p-PYCR1. **P < 0.01 vs sh-NC; ^##^*P* < 0.01 vs miRNA inhibitor-NC; ^ΔΔ^*P* < 0.01 vs vector control

To explore whether circ_0000705 promoted ESCC proline metabolism and malignant phenotype through the miR-621/PYCR1 axis, rescue assays were carried out by decreasing miR-621 expression via transfection with miR-621 inhibitor or increasing PYCR1 expression via transfection with p-PYCR1 in KYSE150 cells with circ_0000705 knockdown. Transwell assays with or without Matrigel showed that the cell invasion and migration were significantly restrained by circ_0000705 knockdown, and then it was partially reversed by miR-621 inhibition or PYCR1 over-expression (Fig. 6A, B).

The reactive oxygen species (ROS) level was significantly increased by circ_0000705 knockdown, and then it was partially reversed by miR-621 inhibition or PYCR1 over-expression (Fig. 6C). Meanwhile, the proline and ATP levels were significantly decreased by circ_0000705 knockdown, and then it was partially reversed by miR-621 inhibition or PYCR1 over-expression (Fig. 6D, E). These results showed that miR-621 inhibition or PYCR1 over-expression partially recovered cell invasion, migration and proline metabolism, which were inhibited by circ_0000705 knockdown.

Consistently, the PYCR1 mRNA and protein expression were significantly suppressed by circ_0000705 knockdown, and then it was partially reversed by miR-621 inhibition or PYCR1 over-expression (Fig. 6F, G). These results showed that miR-621 inhibition or PYCR1 over-expression partially recovered PYCR1 mRNA and protein expression levels, which were inhibited by circ_0000705 knockdown.

### PYCR1 expression is negatively correlated with miR-621 expression, and positively correlated with circ_0000705 in ESCC tissues

**Fig. 7:**
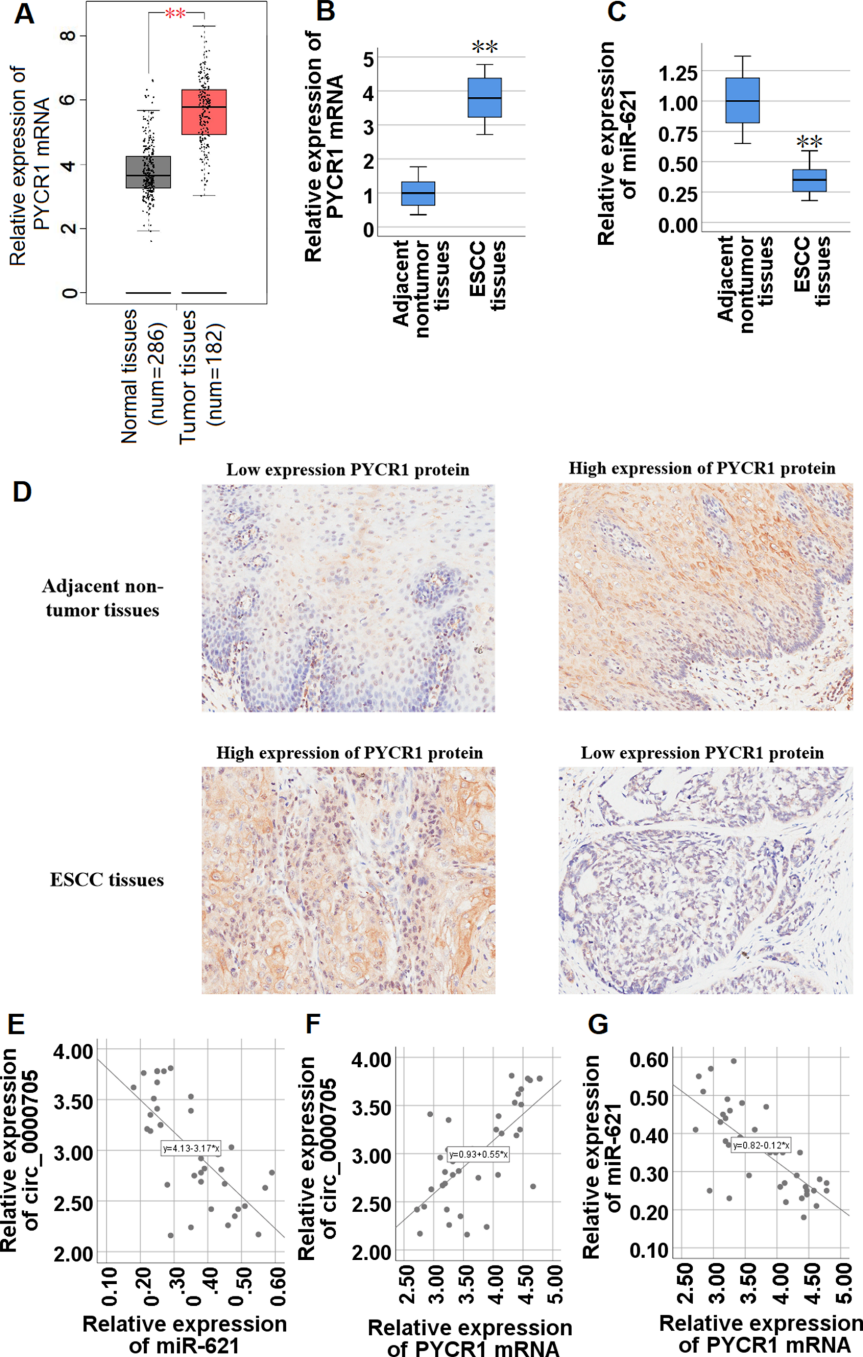
The expression patterns of PYCR1 mRNA and miR-621, and the correlations between circ_0000705 and miR-621 or PYCR1 mRNA in ESCC tissues. **A** The box plot reporter assay reveals the differential expression of PYCR1 mRNA in 286 normal tissues and 182 EC tissues in the GEPIA database. **B** The expression of PYCR1 mRNA was analyzed by qRT-PCR in ESCC tissues and adjacent non-tumor tissues (n = 40). **C** The expression of miR-621 was analyzed by qRT-PCR in 40 ESCC tissues and adjacent non-tumor tissues. **D** Immunohistochemical detection of PYCR1 protein expression in ESCC tissues and adjacent non tumor tissues. The positive staining is brownish yellow and located in the cytoplasm or nucleus (Original magnification × 200). **E** The correlations between circ_0000705 and miR-621 expression levels were analyzed by Pearson’s correlation test in ESCC tissues. **F** The correlations between circ_0000705 and PYCR1 mRNA expression levels were analyzed by Pearson’s correlation test in ESCC tissues. **G** The correlations between miR-621 and PYCR1 mRNA expression levels were analyzed by Pearson’s correlation test in ESCC tissues. *P < 0.05, and **P < 0.01 vs control

To assess the role of PYCR1 in ESCC tissues, we acquired its expression profile in 182 EC tissue samples and 286 normal tissue samples from GEPIA database (http://gepia.cancer-pku.cn/index.html), and found that the mRNA of PYCR1, a key rate limiting enzyme in proline metabolism, was significantly up-regulated in EC tissues compared with normal tissues (Fig. 7A). Subsequently, we validated PYCR1 mRNA expression in tissue samples from 40 patients with ESCC, and confirmed that PYCR1 mRNA was significantly higher in the ESCC tissues compared with adjacent non-tumor tissues (Fig. 7B). miR-621 expression in the 40 specimens of ESCC tissues was also examined by qRT-PCR assay, and the present result demonstrated that, compared with that found in the paired adjacent non-tumor tissues, miR-621 expression was significantly lower in ESCC tissues (Fig. 7C).

Moreover, the results in tissue arrays from 78 ESCC patients showed that the high expression rate of PYCR1 protein in ESCC tissues was 55.13% (43/78), the high expression rate of PYCR1 protein in adjacent non-tumor tissues was 38.46% (30/78), and there was significant difference between the two groups (*P* < 0.05; Table 5). PYCR1 protein was obviously expressed in the cytoplasm and nucleus of ESCC tissues, while a small amount was expressed in the cytoplasm and nucleus of adjacent non-tumor tissues (Fig. 7D).

**Table 5:** Expression and comparison of PYCR1 protein in ESCC and adjacent non-tumor tissues in tissue arrays

Groups	High expression of PYCR1 protein	Low expression PYCR1 protein	*χ*^2^ value	*P* value
ESCC tissues	43	35		
Adjacent non-tumor tissues	30	48	4.351213	0.036983*

*P < 0.05

In ESCC tissues, circ_0000705 expression manifested a negative and significant correlation with miR-621 (r =  − 0.689, *P* < 0.001; Fig. 7E), and a positive and significant correlation with PYCR1 mRNA (r = 0.692, *P* < 0.001; Fig. 7F). In addition, there was a negative and significant correlation between the miR-621 and PYCR1 mRNA expressions in ESCC tissues (r =  − 0.717, *P* < 0.001; Fig. 7G). These results suggested that circ_0000705 negatively regulated miR-621 expression and positively regulated PYCR1 mRNA expression, and meanwhile miR-621 negatively regulated PYCR1 mRNA expression in ESCC tissues.

## Discussion

Although a number of circRNAs are found to be aberrantly expressed and play important roles in ESCC by regulating a variety of malignant biological behaviors, such as cell proliferation, invasion and migration [10–12], no studies have so far addressed circ_0000705’s role and function in ESCC. In the present study, we found that circ_0000705 was significantly up-regulated in ESCC tissues and cell lines, and high circ_0000705 expression was significantly correlated with lymph node metastasis, TNM stage and poor survival. Furthermore, the role and mechanism of circ_0000705 in promoting proline metabolism and progression of ESCC are explored in the present study, and this study demonstrated the tumor-promoting function of circ_0000705 in ESCC by regulating purine metabolism.

Proverbially, metabolic reprogram has been associated with multiple stages of cancer progression, including oncogenic transformation, evasion of apoptosis, tumor metastasis and drug resistance [27–32]. Among changes in tumor metabolism, the role of proline metabolism has gradually received increasing attention [14–16, 28–32]. Increasing evidence has showed that altered proline metabolism exerts a critical effect on cancer cell proliferation [29]. Moreover, elevated proline metabolism facilitates tumor invasion and metastasis, especially by providing more metabolic intermediates for many bio-synthetic pathways [13, 30]. Therefore, targeting proline metabolism may be an effective strategy for prevention and treatment of cancers. During the progression of proline metabolism, the ROS production level is decreased, and meanwhile proline and ATP production levels are increased, which finally facilitates tumor metastasis and invasion [31, 32]. In the present study, we found that circ_0000705 functioned as a promoter of proline metabolism in ESCC cells by facilitating the proline and ATP production and meanwhile restraining ROS production, suggesting that circ_0000705 may facilitate ESCC progression by promoting proline metabolism.

Interestingly, PYCRs, a group of key enzymes in proline metabolism, was found to function as tumor-promoting factors in various cancers [33–35]. PYCR1 inhibition with pargyline lowered intracellular proline level and inhibited cell proliferation of breast cancer cells [34]. And PYCR1 inhibition with Kindlin-2 inhibited proline synthesis and cell proliferation, and increased ROS production and apoptosis in lung adenocarcinoma cells [36]. In the present study, we found that the mRNA expression of PYCR1 was significantly up-regulated in EC tissues via GEPIA (Gene Expression Profiling Interactive Analysis; http://gepia.cancer-pku.cn/) database analysis, which was further confirmed in the detection of our ESCC tissue samples, suggesting that PYCR1-mediated proline metabolism disorders may be involved in ESCC progression.

Recent studies demonstrated that miR-1207-5p and miR-328-3p suppressed the progression of prostate cancer and lung adenocarcinoma by targeting PYCR1 [37, 38]. In the present study, we found that miR-621 manifested a negative and significant correlation with PYCR1 mRNA expression in ESCC tissues, miR-621 over-expression decreased PYCR1 expression, and miR-621 inhibition increased PYCR1 expression in ESCC cells. Thus, based on the above analysis, we speculated that PYCR1 mRNA expression might be down-modulated by miR-621, and PYCR1 was chosen as the potential target gene of miR-621 for our further study. Moreover, recent evidence has manifested that miRNAs regulate their target gene expression by base-pairing with their mRNA 3′-UTR [39]. Here, we demonstrated that PYCR1 mRNA 3′-UTR contains a potential binding site for miR-621 via bioinformatics analysis. Moreover, the luciferase reporter assay verified the binding sites between miR-621 and PYCR1 mRNA 3′-UTR, indicating miR-621 might interact with PYCR1 mRNA 3′-UTR. Therefore, our present results showed that miR-621 could regulate PYCR1 expression by base-pairing with PYCR1 mRNA 3′-UTR in ESCC cells.

Recently increasing studies have confirmed that ceRNA networks based on circRNAs exist widely among various cancers, and it may have an impact on the occurrence and development of various cancers [9]. Moreover, it has been suggested that circRNAs could act as ceRNAs for miRNAs to modulate diverse cell biological processes in ESCC [10–12]. In the present study, the analysis via bioinformatics analysis, Venn diagram analysis and RNA pull-down showed that miR-621 may be one of the potential target molecules of circ_0000705, and PYCR1 mRNA may be one of the potential target molecules of miR-621. Moreover, the RIP, RNA pull-down and luciferase reporter assays verified the relationships between circ_0000705 and miR-621 or PYCR1 mRNA in ESCC cells. Therefore, these above results indicated that circ_0000705 could serve as a ceRNA by sponging miR-621 to promote PYCR1 expression in ESCC cells.

Increasing studies have shown that some circRNAs could play an important role in the metabolisms of amino acids, such as glutamine and serine, by targeting their corresponding miRNAs/target genes axes [18–21]. In the present study, we found that circ_0000705 knockdown decreased PYCR1 expression, proline and ATP production, and meanwhile increased ROS production in ESCC cells. Moreover, our rescue assays indicated that inhibition of miR-621 or restoration of PYCR1 expression reversed the inhibitory effects of circ_0000705 knockdown on PYCR1 expression, proline, ATP and ROS levels in ESCC cells. Therefore, it is feasible that circ_0000705 may facilitate proline metabolism in ESCC cells by targeting miR-621/PYCR1 axis.

## Conclusion

These findings indicated that circ_0000705 could promote proline metabolism and ESCC progression by targeting miR-621/PYCR1 axis, suggest a key role for circ_0000705 in proline metabolism in ESCC, and imply that circ_0000705 may be a potential biomarker or/and possible therapeutic target for ESCC. However, whether circ_0000705 is also highly expressed and has a similar mechanism of regulating proline metabolism in other types of tumors deserve further research in the future.

## Data Availability

The datasets used and/or analyzed during the current study are available from the corresponding authors on reasonable request.

## Abbreviations

circRNA: Circular RNA
ESCC: Esophageal squamous cell carcinoma
PYCR1: Pyrroline-5-carboxylate reductase-1
qRT-PCR: Quantitative real-time PCR
CCK-8: Cell counting kit-8
RIP: RNA immunoprecipitation
ceRNA: Competing endogenous RNA
EC: Esophageal cancer
OSR: Overall survival rate
ncRNA: Non-coding RNA
miRNA: MicroRNA
shRNA: Short hairpin RNA
ROS: Reactive oxygen species
ATP: Adenosine triphosphate
UTR: Untranslated region
TNM: Tumor-node-metastasis
